# Planning for decentralized, simplified prEP: Learnings from potential end users in Ga-Rankuwa, gauteng, South Africa

**DOI:** 10.3389/frph.2022.1081049

**Published:** 2023-01-09

**Authors:** Paballo Mataboge, Susan Nzenze, Nqaba Mthimkhulu, Mbali Mazibuko, Alison Kutywayo, Vusile Butler, Nicolette Naidoo, Saiqa Mullick

**Affiliations:** Faculty of Health Sciences, Wits Reproductive Health and HIV Institute (Wits RHI), University of the Witwatersrand, Johannesburg, South Africa

**Keywords:** hiv prevention, dapivirine vaginal ring, long-acting cabotegravir, decentralized, simplified service delivery

## Abstract

**Background:**

In South Africa, youth experience challenges with oral Pre-Exposure Prophylaxis (PrEP) access and uptake. Taking services out of healthcare settings has the potential to increase reach and overcome these challenges. This paper presents young and older people's preferences for decentralized, simplified PrEP service delivery and new long-acting HIV prevention methods, in Ga-Rankuwa, South Africa.

**Methods:**

Between May and August 2021, both PrEP user and non-user adolescent girls and young women (AGYW), pregnant AGYW, female sex workers, adolescent boys and young men (ABYM), and men who have sex with men (MSM) were recruited to participate in focus group discussions (FDGs) in Ga-Rankuwa, Gauteng. Twenty-two FGDs were conducted. Participants were asked about PrEP uptake, potential acceptability of long-acting HIV prevention products, provision of integrated, simplified, and decentralized services, and digital tools to facilitate access to PrEP and other SRH services. A qualitative approach using inductive thematic analysis was carried out to explore emerging themes on decentralized, simplified delivery and the acceptability of long-acting methods.

**Results:**

Of the 109 participants included in the study approximately 45% (*n* = 50) were female, the median age was 23 years ± 5.3. A third (*n* = 37) were current or previous PrEP users, of which, 59.5% (*n* = 22) collected PrEP refills from the clinic. Decentralized, simplified service delivery was appealing; health facilities, pharmacies and institutions of learning were preferred as service points for PrEP and SRH services, and recreational spaces preferred for dissemination of health information and engagement. ABYM were more open to having recreational spaces as service points. Long-acting Cabotegravir was preferred over the Dapivirine Vaginal Ring due to concerns around perceived side-effects, efficacy, and comfort.

**Conclusion:**

Providing long-acting PrEP methods through decentralized, simplified service delivery was appealing to this population. They provided practical locations for decentralized service provision to potentially increase their engagement with and uptake of HIV prevention and SRH services.

## Introduction

South Africa has the largest HIV epidemic in the world with an estimated 8.3 million people living with HIV and nearly 400,000 new infections per year (1, 2). AGYW aged 15–24 years are at high risk of acquiring HIV, accounting for 25% of new HIV infections each year, three times higher than their male counterparts (3). HIV incidence is also high for pregnant and breastfeeding AGYW and women in South Africa (3, 4).

Women and children have typically been the focus in HIV prevention programs (5). However, engaging adolescent boys and young men (ABYM), and older males, as sexual partners to AGYW, remains a gap in programming to break the cycle of HIV transmission (6, 7). Men who have sex with men (MSM) and female sex workers (FSW) are also disproportionately affected by the HIV epidemic: 18.1% of MSM and 57.7% of FSW in South Africa are living with HIV (8). MSM face high levels of stigma and homophobic violence (9, 10) whilst among FSW, criminalization, stigma and discrimination are barriers to accessing HIV services (11). Programs, therefore, need to provide these populations with easily accessible HIV prevention services, tailored to their needs, seeking to decrease their HIV risk.

The development of biomedical HIV prevention methods, namely oral PrEP, provides the opportunity for at risk populations to take control of their sexual and reproductive health (12). Oral PrEP is the most effective biomedical HIV prevention method that is currently available (13), reducing the risk by more than 95% when taken consistently, as prescribed (13, 14). As of June 2022, there were 626,854 individuals who had been initiated on PrEP in South Africa (15). However, PrEP uptake and continuation remains a challenge in South Africa due to a myriad of factors. These include, lack of support from parents, partners, negative healthcare provider attitudes, confusing PrEP with lifelong antiretroviral treatment (ART) distance to facilities, long clinic waiting times, HIV related stigma, low PrEP awareness, pill burden, forgetting to take PrEP daily, poor HIV risk perception and the lack of youth friendly health facilities (16–25). There are also service delivery challenges to providing PrEP, such as laboratory monitoring which forces users to attend regular clinic visits (26). There are also issues around discomfort with the health system in dealing with sex related issues (26). For instance, cultural and religious norms may cause HCP biases towards sexuality which often results in discriminatory care experienced by clients (19). These biases may affect the clients access to and uptake of PrEP, whereby HCPs with these beliefs and attitudes may be less likely to provide PrEP to potential end users (19). Inadequate clinic preparedness, poor infrastructure, staff capacity, and staff attitude towards key populations have also been noted as challenges to delivering PrEP (27).

Some of these challenges could be overcome by providing differentiated services (28). This involves decentralizing and simplifying services by moving them out of a traditional healthcare setting and expanding the access to services and collection of medication to community pick up points, pharmacies, and community-based organizations (CBOs) (28). A pharmacist-managed PrEP clinic called One-Stop-PrEP in Seattle, United States, was proven to be a successful alternative PrEP delivery model, with high initiation rates, low discontinuation, and loss-to-follow-up rates (29). Self-management models of PrEP delivery in the United States allowed clients to replace in person clinic visits with home-based self-care, through self-testing for HIV or self-sampling for STI's (30, 31). In Tanzania healthcare workers carrying a backpack full of resources conducted routine community HIV testing, ART initiation and refills, and PrEP refills at hotspot sites, improving PrEP continuation for people experiencing challenges in accessing health facilities (32). Providing differentiated services also includes shifting appropriate tasks from nurses to pharmacists, lay providers, and Ward Based Primary Healthcare Outreach teams (33–35) and providing integrated services. A PrEP delivery model led by MSM, and transgender women (TGW) community health workers in Thailand was found to be successful in providing PrEP services to MSM and TGW (36). Given the high burden of HIV and unmet need for contraception among South African women (37), and high rates of sexually transmitted infections (38), decentralizing PrEP and SRH services may also enable programs to better address missed opportunities for providing contraception and STI management. Providing self-care tests such as HIV self-screening kits, human papillomavirus and STI self-sampling kits, pregnancy tests, and making some contraceptive products available over the counter (33, 39) are potential decentralized service options while introducing new PrEP formulations is a simplified service option.

Two long-acting HIV prevention methods, long-acting cabotegravir (CAB-LA) and the Dapivirine vaginal ring (DVR), have been found to be safe and effective (40–42). In March 2022, the DPV ring received regulatory approval from the South African Health Products Regulatory (SAHPRA) for use by women aged 18 and older (43). In December 2022, CAB-LA was approved by SAHPRA for use in people weighing ≥35 kg (44). CAB-LA has also been approved by other countries such as the United States, Australia and Zimbabwe (44). Both these options are not yet available for use in public clinics. The discretion offered by these long-acting methods may result in less stigma associated with PrEP use (45, 46), increase access, uptake and effective use.

We carried out formative research in Ga-Rankuwa, Gauteng, South Africa with two aims. Firstly, to refine our design of decentralized, simplified service delivery utilizing the hub and spoke concept as the foundation (47). In the hub and spoke concept, a primary health care clinic (the hub), is supported by a range of additional community service points for the provision of PrEP and SRH services (spokes). Secondly, we sought to examine end-users' preferences for existing and new long-acting HIV prevention and PrEP methods to simplify PrEP delivery.

The aims of this formative study are important in preparation for expanding services and access to PrEP products. Findings from this formative research will provide insights into the acceptability of decentralized and simplified health service points for PrEP and SRH delivery and provide data on acceptability of new long-acting HIV prevention methods.

## Methods

### Study design and setting

We conducted a qualitative study between May and August 2021 in Ga-Rankuwa, Tshwane district, Gauteng Province, South Africa. Ga-Rankuwa, is home to over 90,000 Black South Africans, it is a peri-urban area, where Setswana is the most spoken language (48). Only 20% of the population are employed and it is politically unstable with frequent, violent service delivery protests (48). Formal housing is in short supply and land invasions have become increasingly common (48)). More than 70% of the population in Ga-Rankuwa is of working age (5–48) and over 50% is female (48). Ga-Rankuwa has three primary health clinics and one community healthcare centre. Oral PrEP is currently available in two of these primary health clinics.

### Study population

The population of interest was AGYW (15–24 years), older women up to the age of 29 years, pregnant AGYW and older women (15–29 years), FSW (>18 years), ABYM, older men up to the age of 29 years, and men who have sex with men (MSM) (15–29 years). ABYM were selected because not only are they at risk of HIV infection and transmission, but they also engage less with HIV prevention services compared to their counterparts (6). MSM and FSW were populations of interest in this study because they are key populations, who are disproportionately affected by HIV compared to men and women in the general population (49–54). Pregnant women are also at increased susceptibility for HIV (4).

### Sampling and recruitment

The study employed purposive sampling to identify the populations of interest. Purposive sampling is the deliberate selection of study participants based on the qualities they possess (55). To recruit female participants, we layered with an existing PrEP implementation programme (Project PrEP^1^), identifying those who had consented to participate in their research related activities. A list of eligible participants (AGYW, ABYM, and women) residing in Ga-Rankuwa, aged 15 −29 years was generated from Project PrEP's database. As Project PrEP focuses on females, to reach MSM, FSW, and the majority of the ABYM a different recruitment strategy was needed. They were recruited through community outreach, facilitated by a CBO in Ga-Rankuwa.

Participants were eligible if they resided in Ga-Rankuwa at the time of the study, were fluent in English, Zulu, and/or Tswana; willing to provide written informed consent, or assent and parental/guardian consent if younger than 18 years. The list included people who were current PrEP users, former PrEP users, repeat PrEP users, as well as those who had never used PrEP. The PrEP user's category enabled us to look at actual experiences with these services. PrEP non-user's participants were considered eligible for PrEP services, therefore may use PrEP, including new long-acting methods, in future, providing information on perceived experiences with these services.

All potential participants were contacted telephonically or approached by trained study fieldworkers. The fieldworkers explained the study and assessed their eligibility, interest, and availability to be part of an FGD. If eligible and willing, they were then invited to participate in the study.

### Study procedures

Two semi-structured FGD guides were developed: one for PrEP non-user participants and one for PrEP users. The aim was to capture differences between PrEP non-users and PrEP users in relation to their experiences, views on long-acting methods and PrEP decision-making.

The PrEP users' FGD guide assessed their journey and the challenges they may have experienced while using PrEP, whilst the PrEP non-users FGD guide examined their knowledge and theoretical acceptability of oral PrEP. Both guides sought to understand their opinions, experiences, and beliefs around the best ways to receive SRH and HIV prevention services. The guides further sought to assess the preferences for a decentralized, simplified service delivery for PrEP and SRH services. In the hub and spoke concept, one Public Health Centre (PHC) is the hub, providing PrEP and SRH services to potential end users. The PHC is supported by, and its footprint expanded through community services points, such as mobile clinics, schools, places where potential end users congregate (such as hair salons, parks, taverns), universities and CBOs. In this study, the service points that were presented to the participants were a hospital, two community healthcare centers, a university campus, ten secondary schools, a police station, a CBO, two pharmacies, a pub and grill, a shopping complex, an eatery, and a park. The service points were identified and selected through prior engagements and end user surveys, where end user's reported socializing. Decentralized and simplified service delivery points were presented to the study participants to facilitate a discussion. Secondly, both guides sought to assess the potential acceptability of long-acting HIV-prevention methods**.** During the workshop, the participants were given a brief description of the new long-acting HIV prevention methods (DVR and LA-CAB), including the mode of delivery and frequency of administration, and the advantages and disadvantages associated with each method. The long-acting cabotegravir injection can be taken by both males and females, while the DVR can only be used by females and those assigned female at birth. Lastly, both guides sought to examine the potential acceptability of demand creation and digital innovations which could facilitate access to information and services. Fieldworkers, two females and one male, were trained on the use of the FGD guides, study procedures including human research ethics, and qualitative interviewing techniques.

Twenty-two FGDs were facilitated between May and August 2021 (Table 1). To encourage engagement during the FGD, participants were categorized into 7 groups based on similar characteristics such as age, sex, sexual preference, namely: “AGYW 15–19 years”, “AGYW 20–29 years”, “pregnant AGYW”, “ABYM 15–24 years”, “ABYM 25–29 years”, “MSM” and “female sex workers”. These groups were further categorized into “PrEP users” and “PrEP non-user participants”. Separate FGDs were conducted as per the abovementioned groups, outlined in Table 1. Following one of the FGD's with PrEP non-user ABYM, we realized that the audio quality of the FGD recording was inadequate. The same participants were called and invited to participate in a repeat FGD, therefore 22 FGDs were completed but only 21 transcripts have been included in the analysis.

**Table 1:** Description of the FGD's.

Population group	Number of FGDs, by PrEP experience			Total number of participants
	PrEP users	PrEP non-users	Total	
AGYW (15–19 years)	2	2	4	17
AGYW (20–29 years)	2	3	5	19
ABYM (15–24 years)	2	2	4	24
ABYM (25–29 years)	2	3	5	22
Female sex workers	1	1	2	10
Pregnant AGYW	0	1	1	4
MSM	0	1	1	8
Unspecified	0	0	0	5
Total	9	13	22	109

COVID-19 protocols were adhered to, including a COVID-19 symptom pre-check, social distancing, sanitizing, and masking. Participants completed a short COVID-19 screening form prior to entering the FGD venue (a large church hall in Ga-Rankuwa). Participants then completed a short, self-administered quantitative survey, collecting demographic information and data on their use and experience of PrEP, social media use and other digital innovations. Following this, FGDs were facilitated by one fieldworker, matched to each group by sex. A second fieldworker took notes during the FGDs. Prior to starting the FGD, participants were reminded that, due to the nature of FDGs, they were not anonymous, but participants were encouraged to keep what was discussed in the group confidential. On average, FGDs were an hour in duration. Using the semi-structured FGD guide, the groups were conducted in English and, where necessary, in participants' preferred language (Setswana, Sotho, or Zulu). The FGDs were audio-recorded on a digital recorder. Participants were assigned a unique number, to ensure anonymity when identifying themselves for the audio recording.

### Data management and analysis

Demographic data were captured on Microsoft Excel and analyzed using Stata v15 (56). FGD recordings were transcribed in the original language and then translated into English, where necessary. The transcripts were reviewed by the research team against the audio recordings to ensure quality checks before being uploaded to a central, secure, access-controlled database. The transcripts were de-identified prior to analysis to ensure participant anonymity. Transcripts were imported and coded in NVivo V12 (57).

Two analysis workshops were held where four research team members (PM, SN, NM, MM) discussed the emerging data, coding, and themes. The first round of codes was created through open coding of 2 transcripts during the workshop. Thereafter, using axial coding, these codes were further organized into 18 main codes. The remaining 19 transcripts were divided among and coded by two research team members (PM and NM). Once all the transcripts were coded, the 18 main codes were further grouped into five major themes, namely, ‘*Dissemination of information’*, ‘*Decentralized and simplified service delivery to facilitate access to services’, ‘Acceptability of long-acting methods’,* ‘*Factors influencing PrEP uptake, effective use, disclosure, and support*’, *and ‘Digital tools*.’ However, this paper only focuses on two themes, namely, ‘*decentralized and simplified service delivery to facilitate access to services’* and ‘a*cceptability of long-acting methods’*.

### Ethical approval and considerations

Ethics approval for the study was granted by the Human Research Ethics Committee of the University of the Witwatersrand (#M210107). Written informed consent/assent was sought from participants before all FGDs, as well as permission to audio-record the discussions. Minors, interested in participation, provided written assent, and provided their parents/guardians phone numbers to the fieldworkers. Parents/guardians of interested minors were contacted telephonically by fieldworkers and informed about the study. Willing parents/guardians gave verbal consent for the minors’ participation in the study.

## Results

A total of 109 participants were recruited (45.9% female, 49.5% male, and 4.8% who identified as ‘Other’). Table 2 provides a description of the participants' demographic characteristics. The median age of the participants was 23 years ± 5.3. Over half of female participants were aged 18–24 years (58.0%). Among the males, there was an equal split between those aged 18–24 years and 25–29 years (42.6% each).

**Table 2:** Participant's characteristics.

Variable	Frequency (%, *n*)			
	Total study population (*N* = 109)	Female (*n* = 50, 45.9%)	Male (*n* = 54, 49.5%)	Other (*n* = 5, 4.8%)
**Age groups**				
15–17 years	7.3 (8)	14.0 (7)	1.9 (1)	0.0 (0)
18–24 years	50.(55)	58.0 (29)	42.6 (23)	60.0 (3)
25–29 years	29.4 (32)	18.0 (9)	42.6 (23)	0.0 (0)
>29 years	8.3 (9)	8.0 (4)	7.4 (4)	20.0 (1)
Missing	4.6 (5)	2.0 (1)	5.6 (3)	20.0 (1)
**Ever used PrEP**	33.9 (37)	38.0 (19)	29.6 (16)	40.0 (2)
No	59.6 (65)	56.0 (28)	63.0 (34)	60.0 (3)
Missing	6.4 (7)	6.0 (3)	7.4 (4)	0.0 (0)
Location of PrEP collection*				
Public health clinic	59.5 (22)	68.4 (13)	60.0 (9)	33 (1)
Mobile van	35.1 (13)	31.6 (6)	33.3 (5)	66 (2)
Private project/Organization	2.7 (1)	0.0 (0)	6.7 (1)	0.0 (0)
Missing	2.7 (1)	0.0 (0)	0.0 (0)	2.7 (0)

With regards to PrEP use, just over a third of all participants (33.9%) had ever used PrEP, higher among females (38.0%) than males (29.6%). PrEP was predominantly collected from a public health clinic (59.5%), followed by mobile outreach services (35.1%), and from private projects/organizations (2.7%).

### Decentralized and simplified service delivery to facilitate access to services

Generally, participants were in favor of decentralized and simplified service delivery, noting the benefits and challenges of providing PrEP and SRH services at each of the presented service points. There was no consensus on which service points the participants preferred the most. The views on each service point are presented in turn below:

a) ***Healthcare Facilities***

Some participants liked the idea of having a local hospital and community healthcare facilities as service delivery points stating that it is “easier to get to the clinic” (ABYM, 25–29 years, PrEP user), they offered a sense of privacy and had friendly staff. However, other participants had opposing views stating that these service delivery points were “far, and it (is) always overcrowded” (AGYW, 15–19 years, PrEP non-user). AGYW highlighted the lack of privacy at health care facilities, opening times, and healthcare provider attitudes as challenges to accessing services:

“*There is no privacy. People enter as they please, so you end up not being able to talk*.” (AGYW, 20 −29 years, PrEP User)

“*Pharmacy is closed, why do you came at this time? PrEP providers are at the back.” I arrive when I arrive there, “Yoh sister why did you come so late? Come tomorrow at eight!” They do not even want to hear my story that I have a file; I am only late for collection. How about they look at my file and give me pills so that I can leave?*” (AGYW, 20–29 years, PrEP User).

The integration of PrEP services with family planning, HIV testing, and other SRH related services was preferred by all participants as this would encourage participants to access holistic prevention services and would reduce the number of clinic visits and money spent on transportation:

“*Yeah, it should be one stop centre so that when I go for family planning then I collect…. [Currently] you have to go there then you go to another place then come back*.” (Female sex worker, 18–29, PrEP user)

“*For instance, I will be going to the clinic, and it's money (meaning that they will use money) …So it's better if you go once to collect PrEP and everything else*.” (AGYW, 20–29 years, PrEP users)

There were a variety of service delivery characteristics that AGYW thought would encourage them to access healthcare services at clinics. These included friendly staff, privacy, shorter waiting times, separate queues for medication pick-ups, and pre-packaged medication (which would reduce time spent in facilities).

“*I will need…yes, the service…Someone who will help you with love and not in a hurry. You know that you will not get reluctant to go to the clinic because you are worried that you will meet a certain kind of person (referring to an unkind healthcare worker). I [need to know] know that the person who is assisting me is friendly*.” (AGYW, 20–29 years, PrEP user)

“*I cannot always travel to (township name) to collect the pills and I am also not sure what time I will come back, maybe around 13h00, because hai! When you go to the clinic to collect medication you queue with everyone, when you go inside to see sisters, whether you only came to collect pills only or not. You queue just like everybody*.” (AGYW, 15–19 years, PrEP user)

b) ***Local police Station***

Participants had mixed views about the local police station as a service delivery point. A few noted that they would not mind receiving SRH and PrEP services at the police station and noted safety and accessibility as reasons for this. The police station was also viewed as a place of respect and dignity.

“*Yah, police station is okay you know why?… Police Station is a place that is being respectable. …And dignity*” (AGYW, 20–29 years, PrEP non-user)

Some participants stated that using the local police station as a service point removed the element of privacy and discretion they would want when accessing services. They also believed that the police officers would foster and perpetuate PrEP related stigma in the community.

“*Those police will judge you if you go there…they talk too much*.” (AGYW, 20–29 years, PrEP User)

However, they were open to considering it if there was a private consultation room. There were no differences between AGYW and ABYM preferences for police stations as service delivery points.

c) ***Local pubs and local eateries***

AGYW and female sex workers stated that places like pubs and eateries would not work as service points because of lack of privacy. They further stated that they feared being judged by patrons if seen accessing PrEP and SRH services at these service points:

“*Mostly you will find drunkards who talk as they wish. So, with us, they may end up saying hurtful words to you*.” (Female Sex Worker, 18–29 years, PrEP user).

They were also concerned about safety and confidentiality at a pub, stating that intoxicated pub-goers will be disruptive towards the healthcare providers and clients seeking PrEP and SRH services. Distance was also noted as a reason against pubs as service points, especially among participants who lived far from the pub on the decentralized and simplified service delivery model. Furthermore, a few AGYW and MSM participants described pubs as inappropriate for receiving PrEP services because pub goers are there to have fun and drink alcohol and won’t want to be told about PrEP.

“*We are going to drink (alcohol) not to take pills*” (AGYW, 20–29 years, PrEP User).

“*I disagree with the pub…Then there you are; “Hey girl come here, PrEP*”*. When am I going to drink? When am I going to drink? No, I came to drink*.” (MSM, 15–29 years, PrEP non-user)

However, one MSM participant expressed that providing self-care products such as lubricants at pubs may draw their attention and encourage their engagement with PrEP and SRH services.

“I feel that if maybe you are at certain, like just do a mini, a mini takeaway thing, it's a PrEP paper [paper bag] maybe you…maybe put in 4 lubes. After putting in 4 lubes I come [to you]. I know that I don’t have lube…Guys also like lube. Which guy doesn’t like lube?… So, you have given us a nice small package friend and we’ll be there.” (MSM, 15–29 years, PrEP non-users). Most ABYM participants were more open to the idea of using pubs and eateries as service delivery points compared with AGYW, MSM and female sex workers. One participant believed that there won’t be any judgement or stigma at the pub because everyone will be there to enjoy themselves and mind their own business.

“*Me I feel like at [name of local pub] there would hardly be any stigma, ‘cos like it's a place where it's by drunkards. Everybody is minding their own business so yes…That's why I feel that for me [it's] comfortable to get them there because no one is gonna judge me*.” (ABYM, 20–29 years, PrEP User)

Lastly, some participants suggested that pubs and local eateries be used to distribute information on PrEP and SRH services and as PrEP pick-up points.

d) ***Local parks***

Female sex workers, MSM and ABYM were open to the park acting as a service delivery point.

“*It is at the park why not? Like you will find that they put up a tent, right?…Yeah, we will enter, collect, and leave*.” (Female sex workers, 18–29 years, PrEP user).

It was noted that parks are good places to access young people, especially on weekends:

“*I prefer like public parks your community parks uh parks. Like there are a lot of people especially like during weekends…Like uh this young generation, our age- I can say uh from eighteen to like twenty-five twenty-six*.” (ABYM, 25–29 years, PrEP user).

They further stated that having events at the park, targeted at educating the community on PrEP and SRH services would attract potential end users.

“*As soon as you do an event at the park, they will say ‘*’*at the park, we are going there friend*”.” (MSM, 15–29 years, PrEP non-user)

However, the issue of safety at parks was raised by AGYW participants with some stating that “It is not safe” (AGYW, 15–29 years PrEP non-user)

“*So, what if we go there and they rob us?*” (AGYW, 15–19 years, PrEP user).

In spite of this, AGYW stated that this could be mitigated if a mobile van and security were provided.

“*[T]hat's why I am saying we need mobiles. Because of once we have mobile at (name of local park) obviously there should be security…So, I think it will only be safe if we have security*.” (AGYW, 15–19-years, PrEP user)

e) ***Schools and universities***

Participants had mixed views about having the local university and secondary schools as service delivery points. The majority of participants felt universities would be acceptable, because they believed that they won’t be judged or stigmatized. They described the university students as understanding and mature, and believed that they too will benefit from the PrEP and SRH services.

“*It would work [be]cause yeah, and a lot of students attending there may consider taking PrEP*.” (Female sex worker, 18–29 years, PrEP user)

“*I think [university name] would also work for me*” (Pregnant AGYW, 15–29 years, PrEP non-user)

Older AGYW and ABYM, pregnant AGYW, female sex workers and MSM were accepting of secondary schools as service delivery points. They felt that secondary schools were safe because:

“*the security at the gate will make sure that you are safe inside the school*” (ABYM, 25–29 years, PrEP non-user).

Older participants also suggested that all schools in Ga-Rankuwa be considered as service points because every zone has a school, thus improving accessibility. They believed that this would ensure that everyone has easy access to PrEP and SRH services and will not need to travel to collect PrEP.

“*So that, when you say it's every school then people can go to all of the schools when we’re in Ga-Rankuwa [to access PrEP services] coz some people are far from [school name], they are far from [it] so…So it will be closer for them. Coz every zone has a school in it so there's no they don’t need to take taxis to go and take the pill*.” (ABYM, 25–29 years, PrEP non-user)

They felt that they would be less likely to experience judgement or stigma in these environments because secondary school children were too young to understand the services they would be seeking.

“*They [young children] know nothing…yes, my mother came to school, doing what? They do not know*.” (AGYW, 20–29 years, PrEP non-user).

However, younger AGYW and ABYM participants were concerned about lack of privacy, stigma and judgement at the secondary schools. They feared that if seen accessing PrEP and SRH services, teachers, security guards and students would judge them.

“*Teachers will judge you at schools [and say] “you are sleeping around, you are sleeping around*””. (AGYW, 15–19 years, PrEP user)

“*I find it's great but at the uh…at schools, eish, mostly we can’t use classrooms because [with] classrooms, anybody is able to come in anytime, it won’t be confidential*”. (ABYM, 15–24 years, PrEP user)

f) ***Pharmacies***

The majority of participants liked the idea of having local pharmacies as service delivery points. They also suggested using private pharmacies at the local shopping complex as places to collect their medication and get multi-month dosing.

“*If they could come up maybe with an action plan so that we can collect them at Dischem [pharmacy chain] you go with a card [and] collect your meds there… I think it could work* (AGYW, 20–29 years, PrEP user).

Participants described pharmacies as convenient, private, and safe service delivery points, with knowledgeable and friendly staff.

“… *pharmacies are the safest places, not the pubs*.” (AGYW, 20–29 years, PrEP non-user)

“*And then also people that work there, they know medication and [how] to treat patients…You arrive, they give you medication, you leave. …No judgements*.” (AGYW, 20–29 years, PrEP user)

g) ***Additional service delivery points and suggestions***

Overall, most participants suggested that the community service points should be private, easily accessible, and close to main transport routes. Participants suggested additional possible service delivery points, including local libraries, community-based organizations (CBO's), community centers, local churches, and brothels. They suggested that local libraries and churches be used for sharing information on PrEP and SRH services, while CBO's, community centers and brothels (specific to FSW) be used for providing PrEP and SRH services.

## Preferences for long-acting methods

Participants were excited about the prospect of having multiple PrEP methods to choose from, based on their needs and preferences. Overall, LA-CAB was preferred compared to the DVR. Most of the participants believed that the long-acting methods would reduce time spent at clinics, eliminate overcrowding at clinics, and ensure method continuation, as they would remove the burden of taking a pill daily (as with oral PrEP). The participants emphasized that their potential uptake of the long-acting methods, once available, would be based on their personal preference and perceived effectiveness of the methods. Their preference was largely based on continuation, convenience, product related issues, and availability of information. No distinct gendered differences were observed.

Specific feedback on preferences, and concerns of the DVR and LA-CAB are detailed below:

a) ***Dapivirine Ring (DVR)***

Most AGYW and all the pregnant AGYW and female sex worker participants stated that they would not use the DVR because of the perceived side effects: and false concerns related to misunderstandings of their anatomy “What if it goes to your womb?” (AGYW, 20–29 years, PrEP user). A few ABYM participants were also worried about perceived side effects, particularly pain during sexual intercourse, and low efficacy of the DVR: “Eventually at the end she will say “ah my guy, I*’*m no longer enjoying sex” then it will become a problem, the relationship will be somehow.” (ABYM, 20–29 years, PrEP non-user).

Some AGYW and female sex worker participants also had concerns about the impact of everyday use of the DVR, using the ring during menstruation and vaginal hygiene. They also expressed concerns around the possibility of the ring being felt during sexual intercourse with one participant stating that; “Coz you know those things right, they can be felt in here (referring to the vagina). And then when you start being intimate with someone, they will feel that thing and it hurts them.” (Female sex worker, 18–29 years, PrEP non-user). The participants were also concerned that the ring will be invasive, and that they might feel discomfort or pain while inserting it.

“*Ai, the ring method… I think it will be painful when I insert something inside my vagina and you are saying you also experience pain during sex, you understand me?*” (**Female Sex Worker, 18–29 years, PrEP user**). *However, few AGYW participants were open to using the DVR once it becomes available highlighting that clear information and no side effects, would allow them to consider using it*.

“*Yeah, for me, as long as it does not cause irritation, like as long as it does not irritate me and remains intact*.” (AGYW, 15–19 years, PrEP user)

b) ***Long-acting Cabotegravir (LA-CAB)***

Most of the participants stated that LA-CAB would be accepted by potential end users and the larger community because of their familiarity with the injectable contraceptive.

“*Uhm (yes), they will accept the injection. It is common, right, for prevention. It is familiar…many people will accept it…they will prefer it*.” (**AGYW, 20–29 years, PrEP user**)

Furthermore, the LA-CAB as an injectable was preferred “Injection is better” (Pregnant AGYW, 15–29 years, PrEP non-user) to the oral pill because it 1) eliminates burden of daily pill taking, 2) ensures continuation because participants will not forget to take pills, and 3) is convenient and discreet to use.

“*I mean, like, you will not have problem with forgetting the pills and whatnot. As long as you…injected*.” (**Female Sex Worker, 18–29 years, PrEP user**)

“But you see once they injected you at least you will not have to stress about taking pills” (**AGYW, 20–29 years, PrEP User**)

“*And the fact that it's discreet*” (**ABYM, 15–24 years, PrEP non-user**)

However, a few AGYW noted that they would not opt for LA-CAB because they feared that they would experience similar side-effects to the injectable contraceptive, such as menstruation cessation and weight gain.

“*Let it be rolled out first because you would inject and gain weight…or it ceases your menstruation I am afraid of [that]*.” (**AGYW, 20–29 years, PrEP user**)

Some also expressed fear of injections:

“*Injection it's just me, I*’*m scared of it*.” (**ABYM, 25–29 years, PrEP user**)

c) ***Alternative suggested methods:***

Despite participants' enthusiasm about the long-acting methods, some participants stated a preference for alternative prevention methods. One PrEP non-user ABYM said he preferred the oral pill instead of LA-CAB because he feared injections and advised that future biomedical inventions should consider developing a once monthly oral PrEP pill, as it would be more convenient and reduce the pill-taking burden.

“*But monthly you can go to the clinic, maybe they can give you a 12 pack for the whole year. So, you only go to the clinic once*” (**ABYM, 20–29 years, PrEP non-user**)

An ABYM participant in a sero-discordant relationship stated that he preferred the daily oral PrEP pills because they did not use condoms. He noted that taking a daily pill is a constant reminder to prevent HIV transmission, and that by taking a pill everyday he could support his partner who was on ART.

“*I*’*m living that life where I*’*m exposed to HIV, so I don*’*t take chances. Oh, uhm, uh I*’*d prefer, I*’*d stay with my pill. Why? Because I*’*m 100% sure of what's happening… Because I*’*m living this life of [being with] an HIV positive partner and we don*’*t use protection, but she is on her pills, and I am on PrEP. So, I honestly will stick to the pills so we*''*ll drink the pills together, … I wouldn*’*t want to do the injection or any of those things. Because I think the pill, they are 90% [effective] and she's also taking her ARVs, her CD4 is still normal*” (**ABYM, 25–29 years, PrEP User**)

A few AGYW participants indicated they would be interested in a multipurpose prevention technology that would prevent HIV and other STI's:

“*Why doesn*’*t the PrEP project bring substances that protect against STIs and other viruses? …how about maybe they add contents that will prevent STIs, and it becomes one*.” (**AGYW, 20–29 years, PrEP User**).

## Discussion

This study sought to assess the acceptability of decentralized, simplified PrEP and SRH service delivery and to explore the range of additional non-facility service delivery sites and user provider interfaces. This study also sought to examine potential end users' preferences for existing and new long-acting HIV prevention and PrEP methods as a means to simplify PrEP delivery. The integration of PrEP and SRH services was valued by participants as this may reduce transport cost and minimize the number of clinic visits. Overall, all participants were accepting of decentralized and simplified service delivery. They shared the pros and cons of each service point and there was no consensus on which service points sub-groups preferred the most. They welcomed the prospect of having more PrEP methods to choose from in the future, however, LA-CAB was preferred over the DVR among most cis women.

Healthcare facilities, pharmacies and schools were generally found to be largely acceptable to the study participants and were seen as suitable for providing full PrEP and SRH services. The police station, pubs, eateries and parks were largely said to be better suited for information dissemination and as PrEP collection points. Taking healthcare out of the hospital or clinic using community-based mobile clinics specialized in HIV services is known to increase access to healthcare and improve health outcomes of patients (12, 58–61). Studies have shown that it is feasible to use pharmacies to dispense ART and other chronic medication (62–64), with a study in Nigeria finding a higher ART continuity rate and viral load suppression in people who utilized pharmacies for refills compared to healthcare facilities (62). There has also been a proposal in South Africa for Pharmacy Initiated ART (PIMART), which will also include PrEP initiations (65). Previous studies the United States of America (United States) and Sub-Saharan Africa also found that actual and potential clients where in support of pharmacy PrEP distribution (66–69). Delivering PrEP through pharmacies has been used to improve accessibility to PrEP in the United States (70).

Preventing HIV infection among ABYM and older men with the goal of reducing HIV transmission to their partners is a key strategy in preventing HIV infection among AGYW (7). However, men in South Africa are less likely to engage with HIV prevention and treatment services (71). Also, healthcare facilities have achieved limited HIV testing, prevention and treatment among men with barriers including distance to the healthcare facility, inconvenient operation hours and confidentiality concerns (71). The acceptability of decentralized, simplified service delivery points among ABYM has the potential to increase the engagement of men with HIV prevention and SRH services and may subsequently lead to a decrease in new HIV infections. Furthermore, compared to AGYW, MSM and FSW's, ABYM in this study were more open to the idea of using pubs, eateries, and parks as service points for PrEP and SRH related services. Published data show that men prefer to receive HIV services outside of the healthcare facility, this may be in the workplace, hotspots where men congregate and places that are easily accessible to men and have flexible operating hours to cater for their busy schedules (71–74). Furthermore, men have reported experiencing healthcare facilities as unwelcoming to them and less responsive to their healthcare needs (75–77). Decentralized and simplified service delivery points should be tailored to also be male-friendly.

Community based social activities have also been found to be viable in reaching men and promoting engagement with HIV services (78, 79). This strategy was also suggested by the study participants as a possible way to attract potential end users and educate them on PrEP and SRH services. Overall, decentralizing and simplifying HIV prevention and SRH services by providing an increased number of service points in the community and providing the option to receive services from non-clinical providers has the potential to increase access to services, and improve clinical outcomes, especially for men. Furthermore, the provision of services needs to be holistic addressing all SRH needs as opposed to just PrEP. This removes stigma and fosters an enabling environment for positive health seeking behaviour. Stigma should be addressed as a potential barrier to decentralised service delivery through community engagement and continuous sensitization (80). Participants described characteristics that influenced the acceptability of each service point, including friendly staff, safe environment distance to the service point, privacy and confidentiality when using the services, and non-judgmental attitudes of other community members Similar to previous research, friendly staff, shorter waiting time, and privacy were noted to be important service delivery characteristics (12, 81, 82). When providers are non-judgmental, sensitive, maintain confidentiality, are supportive and empathetic, end users are more likely to seek and benefit from healthcare services (83). Other service delivery points suggested by participants included the local libraries, community-based organizations (CBO's), community centers, local churches, and brothels. Despite having a range of participant groups represented in this research, there was no group consensus around a specific preferred service delivery point: this highlights the need for a range of service points to meet the needs of a variety of populations.

Participants believed that the long-acting methods would reduce the pill-taking burden and allow for the discreet use of an HIV prevention method. They also believed that long-acting methods would increase PrEP continuation. These views are supported by findings from LA-CAB and DVR clinical trials (84, 85). For chronic conditions, long-acting medication has improved medication adherence when compared to daily oral medication, which has led to improved clinical outcomes (86, 87). The LA-CAB method was preferred over the DVR. LA-CAB side-effects were perceived to be brief and easier to manage, and participants were more familiar with injectables. With regards to the DVR, the AGYW and female sex workers were concerned about product efficacy, discomfort or feeling pain during ring insertion, potential impact on their sex lives and intimate relationships, using the ring during menses, and perceived side effects. ABYM raised similar concerns about the ring, particularly around the anticipated side effects and discomfort during sexual intercourse. This is a common concern that has arisen in other studies exploring men and women's sexual experience while using the DVR in Malawi, South Africa, Uganda, and Zimbabwe (88, 89). However, in these studies, most men reported that the ring did not affect their sexual positions, frequency, or their experience of sexual intercourse, while female partners reported that the ring made the vaginal environment more desirable (88, 89). Furthermore, studies have found that increased duration of DVR use is associated with acceptability, ease of use, comfortability during sex (90–92) and continued use of the ring during menstruation (93). This reinforces the need to engage potential users, their partners, and the community to address DVR hesitancy, as well as concerns around the ring and its effect on sexual encounters. This may result to better acceptability of the ring. Positive messaging and data from first-hand accounts among experienced ring users would also be beneficial. In addition, if decisions are made to use the DVR covertly, the risk of ring discovery should be discussed with potential users to avoid the potential for distrust and negative reactions from their partners (89). A few AGYWs indicated their interest in a prevention method, preventing HIV and other STI's. Multipurpose prevention technologies (MPT) are prevention products that simultaneously offer protection against HIV, other STI's and/or unintended pregnancy (94). There is a growing array of MPT candidates in development (95). Combining prevention approaches to address unplanned pregnancy, HIV and STI's offers a unique opportunity to reduce cost, improve adherence and improve sexual health (95).

One strength of this study was that it included perspectives from a wide range of potential end users. The study also contributes to formative evidence that may inform the design of decentralized delivery. There are also some limitations. The service delivery points were specific to the study area, so challenges to certain delivery points, like distance and transport costs may be specific to this area, therefore, the findings are not generalizable to other areas. While the techniques for data saturation (96) were not formally used during data collection, during the analysis, no new themes emerged, therefore data saturation was achieved.

## Conclusion

There remain gaps in access, uptake, and effective use of HIV prevention and SRH services among target populations. Decentralizing and simplifying PrEP and SRH services by providing services outside the traditional healthcare setting will bring services closer to clients and has the potential to increase access to and engagement with end users, especially men. Furthermore, service orientation and delivery cannot be considered homogenous. Different population groups have different needs and preferences; therefore, they need service delivery options that provide integrated services and fit their preferences and lifestyle.

Addressing concerns of the DVR through counselling and sex positive messaging could improve its potential acceptability among cis women. A variety of engagement and education strategies with potential users, partners and key gate keepers are also critical to the uptake and effective use of any HIV prevention methods. Nonetheless, increased choice in HIV prevention methods allows the end user to choose a method that meets their needs and suits their lifestyle. Providing a variety of long-acting PrEP methods, has the potential to increase access to prevention services, subsequently leading to increased uptake, improved continuation, and a decrease in new HIV infections.

## Data Availability

The raw data supporting the conclusions of this article will be made available by the authors, without undue reservation.

## Footnotes

^1^Project PrEP, funded by Unitaid and led by Wits Reproductive Health and HIV Institute, is an AGYW-focused implementation science project seeking to inform the introduction and integration of oral PrEP as part of combination HIV prevention and sexual and reproductive health services in South Africa. Approval for the implementation of this study was received from the Wits Human Research Ethics Committee (M180860).
